# Supplementary material for: Progressive alterations in polysomal architecture and activation of ribosome stalling relief factors in a mouse model of Huntington’s disease
Source: Neurobiol Dis. Author manuscript; Available in PMC 2024 Jul 22. (PMC7616275; doi:10.1016/j.nbd.2024.106488)
Supplement: Appendix A. Supplementary data [file EMS197566-supplement-Appendix_A__Supplementary_data.pdf]

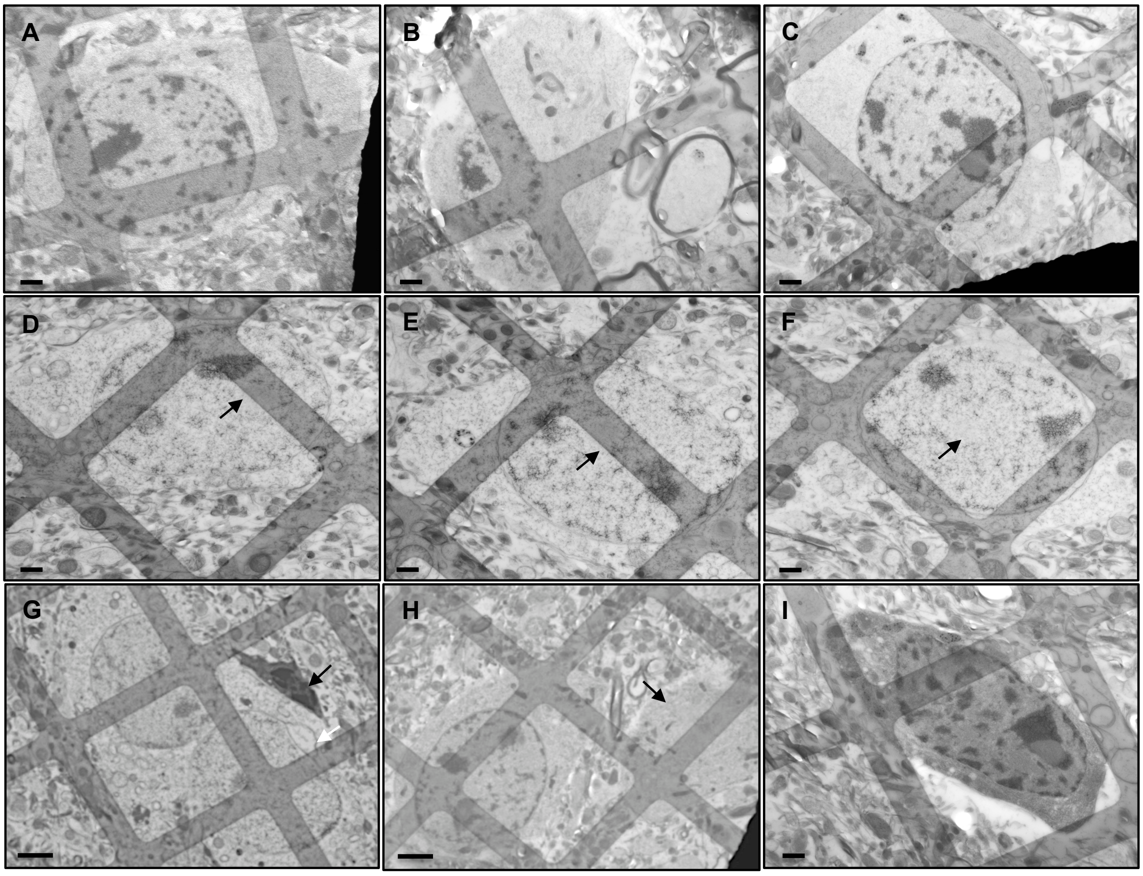

Supplementary Figure S1. Representative EM images of striatal cells from 250-nm-thick sections of brain tissue. (A-F) Cells compatible with medium-sized spiny neurons (MSSN) based on morphological criteria in wt control **(A-C)** and heterozygous zQ175 **(D-F)** mice aged 11 months. In the HD model, these cells appear paler, an effect evident in the chromatin, nucleus and cytoplasm. In addition, nuclear inclusions are identifiable in the HD model (arrows in D-F). **(G)** A cell, likely microglia, with nucleus and cytoplasm that are highly dense (black arrow) is observed near two pale cells compatible with MSSNs. One of these MSSNs contains a nuclear bleb (white arrow), which are often found in HD neurons and are indicative of neurodegeneration. **(H)** A putative MSSN and, on the right, an area of cytoplasm from another cell (arrow) with a similar appearance but not showing the nucleus. **(I)** A cell from a control animal, with size and morphology compatible with a MSSN, with highly dense nucleus and cytoplasm (signs of cell death). Scale bars: 1 µm (A-F, I), 2 µm (G-H).

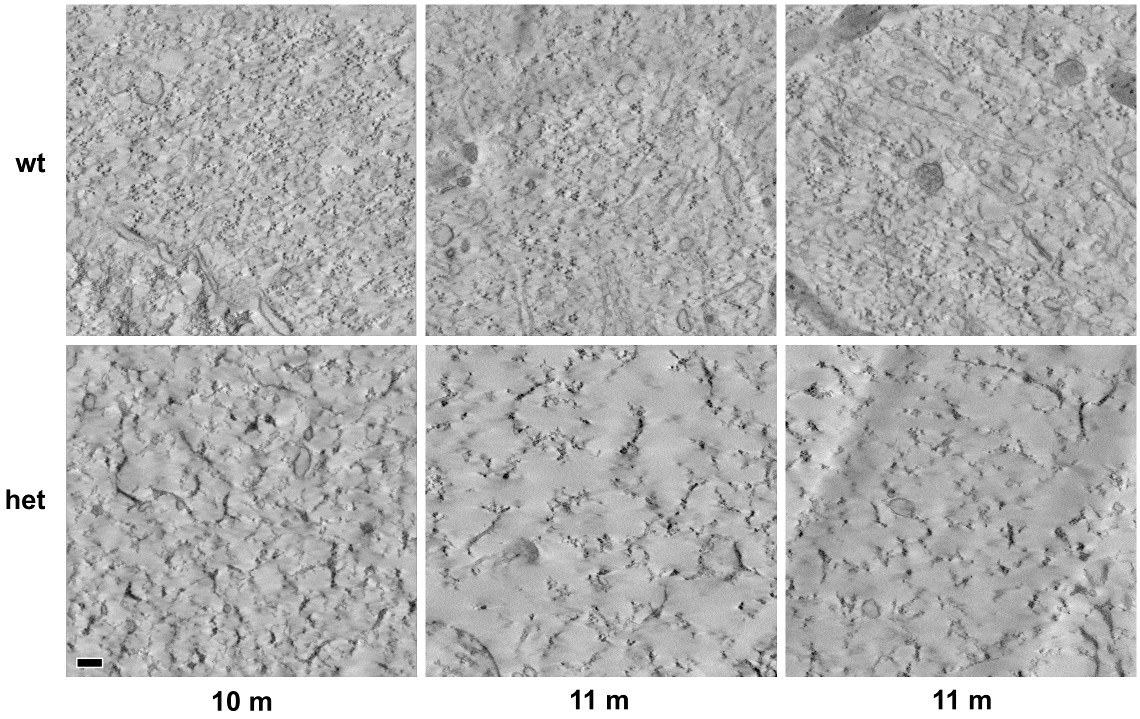

Supplementary Figure S2. Representative slices of tomograms from 10 and 11-month-old wild-type control (top) and heterozygous zQ175 (bottom) mice. Scale bar: 200 nm.

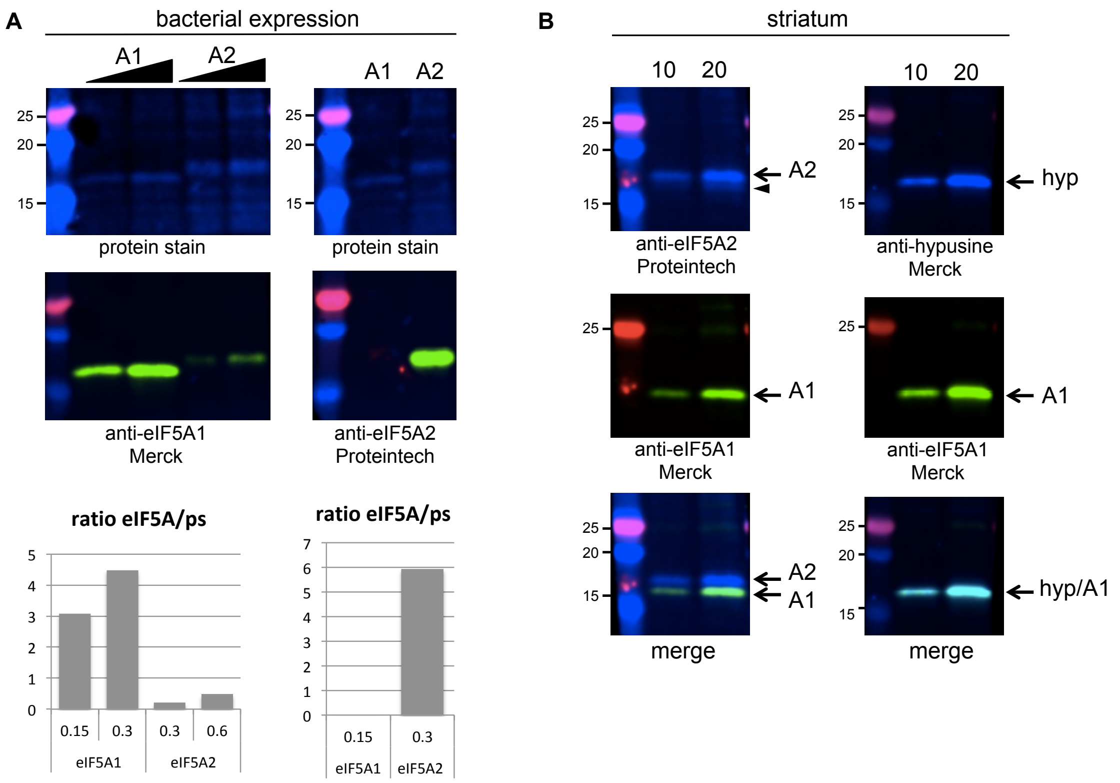

Supplementary Figure S3. Specificity test of antibodies for eIF5A1 and eIF5A2. (A) The sequences coding for human eIF5A1 and eIF5A2 were amplified from the Human MTC Panel I (#636742, Clontech) and cloned in pRSET-B vector using NdeI and EcoRI sites and overexpressed in *Escherichia coli* C41 cells. Upper panels show total protein accumulation in C41 cells transformed with the corresponding plasmid either pRSET-eIF5A1 or pRSET-eIF5A2 (Revert™ 700 Total Protein Stain, 926-11021, LI-COR). Overexpressed eIF5A1 has a higher mobility than eIF5A2. Lower panels show the incubation of the above membranes with the corresponding antibodies. The mouse monoclonal antibody raised against eIF5A1 (SAB1402762, Merck) recognizes both proteins but preferentially recognizes eIF5A1. The quantification of the relative intensity normalized by the amount of the specific protein (protein stain of the corresponding band) showed that the antibody recognizes eIF5A1 approximately 10 times better than eIF5A2. Meanwhile, the rabbit polyclonal raised against eIF5A2 (17069-1-AP, Proteintech) efficiently recognizes eIF5A2 while very poorly recognizes eIF5A1 (the band is only visible if the membrane is overexposed). The quantification of the relatively intensity normalized to the amount of each corresponding protein shows that the antibody raised against eIF5A2 recognizes eIF5A2 200 times better than eIF5A1. See graphs on the bottom panels. **(B)** We evaluated the performance of these two antibodies in mouse striatal samples. Left-upper panel shows that the antibody against eIF5A2 recognizes a band of the expected size and a shadow band of higher mobility (arrow head, likely eIF5A1). The panel below shows that the antibody

raised against eIF5A1 only recognizes a band of the expected size for eIF5A1 in the striatum. The merge of both channels (left-bottom panel) confirms that both proteins can be detected at the same time in the striatum and also confirms the higher mobility of eIF5A1 in striatal samples. These results also suggest, together with the quantifications made in (A) that the accumulation of eIF5A2 protein is lower than that of eIF5A1 in the striatum. On the right-upper panel we observed that the incubation of striatal samples with polyclonal anti-hypusine antibody (ABS1064-I, Merck) detects a single band and the co-incubation with mouse monoclonal antibody raised against eIF5A1 (SAB1402762, Merck) confirms that this band corresponds to eIF5A1 (merge bottom panel). Thus we cannot detect hypusinated eIF5A2 in the striatum, either because eIF5A2 does not have this modification or because the amount of hypusinated eIF5A2 does not reach the detection limits for the anti-hypusine antibody.
